# An LCK/PPARG/SCD Lipid Metabolism Axis Promotes Invasion in Human Oral Cancer

**DOI:** 10.1002/mco2.71060

**Published:** 2026-10-13

**Authors:** Jonas Pyko, Tamara Tomin, Markus Glaß, Lisa Müller, Jana Macho, Matthias Schittmayer, Monika Haemmerle, Ruth Birner‐Gruenberger, Tony Gutschner

**Affiliations:** ^1^ Section for RNA Biology and Pathogenesis Institute of Molecular Medicine Faculty of Medicine Martin Luther University Halle‐Wittenberg Halle (Saale) Germany; ^2^ Section for Bioanalytics Institute of Chemical Technologies and Analytics Faculty of Technical Chemistry TU Wien Vienna Austria; ^3^ Section for Molecular Cell Biology Institute of Molecular Medicine Faculty of Medicine Martin Luther University Halle‐Wittenberg Halle (Saale) Germany; ^4^ Section for Experimental Pathology Institute of Pathology Faculty of Medicine Martin Luther University Halle‐Wittenberg Halle (Saale) Germany; ^5^ Diagnostic and Research Institute of Pathology Medical University of Graz Graz Austria

**Keywords:** head and neck squamous cell carcinoma, invasiveness, lipid droplets, oral squamous cell carcinoma, phospholipid composition

## Abstract

Oral squamous cell carcinoma (OSCC) frequently metastasizes to regional lymph nodes, yet the metabolic adaptations that enable invasive dissemination remain incompletely understood. Although lipid metabolic reprogramming has emerged as a hallmark of metastatic dissemination, the upstream signaling pathways coordinating these processes remain poorly defined. Here, we combined transcriptomic, epigenomic, and lipidomic analyses with genetic and pharmacological perturbation of lymphocyte cell‐specific protein tyrosine kinase (LCK) and peroxisome proliferator‐activated receptor gamma (PPARG) to define the signaling network linking oncogenic kinase activity to lipid metabolism. We identify PPARG as a critical downstream effector of LCK that promotes invasion by reprogramming lipid metabolism. Mechanistically, LCK maintained PPARG expression, which induced stearoyl‐CoA desaturase (SCD), increased monounsaturated fatty acid synthesis, and limited lipid peroxide accumulation. Pharmacological inhibition of SCD suppressed cancer cell invasion, impaired viability, and sensitized PPARG‐activated cells to ferroptosis‐associated cell death. Together, these findings establish an LCK/PPARG/SCD signaling axis that couples oncogenic signaling to lipid metabolic plasticity during OSCC invasion. Our study identifies SCD as a potential therapeutic vulnerability and suggests that therapeutic activation of PPARG signaling should be approached with caution in patients with established oral cancer and carefully evaluated in biomarker‐guided clinical studies.

## Graphical abstract

Oral squamous cell carcinoma (OSCC) progression is driven by invasive tumor‐cell phenotypes, for which effective targeted therapies remain limited. Here, we identify a tumor‐cell‐intrinsic LCK/PPARG/SCD axis, in which LCK regulates PPARG expression and H3K27 acetylation, while PPARG promotes lipid remodeling and SCD‐dependent fatty acid desaturation to support OSCC invasion and protect against lipid peroxidation. These findings reveal a context‐dependent metabolic vulnerability that may provide a basis for targeting SCD in LCK/PPARG‐active OSCC.

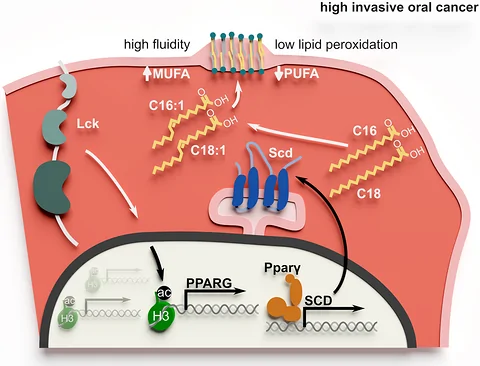

## 1 Introduction

Oral squamous cell carcinoma (OSCC), the most common malignancy of the head and neck region, arises at several anatomical subsites. Major risk factors include excessive alcohol and tobacco consumption, areca nut chewing, and human papillomavirus (HPV) infections. According to GLOBOCAN 2022, cancers of the lip and oral cavity account for 389,485 new cases and 188,230 deaths annually [1]. Despite advances in diagnosis and treatment, the 5‐year overall survival (OS) rate remains at approximately 65%. Lymph node metastasis (LNM) is a major prognostic factor, with a 5‐year survival rate of 23%–63% in patients with LNM (N1–3, LNM^pos^) compared with approximately 74% in patients without LNMs (N0, LNM^neg^) [2]. Understanding the molecular mechanisms driving OSCC invasion and metastasis is therefore essential for identifying biomarkers and therapeutic vulnerabilities.

Recent studies have highlighted the contribution of lipids to oral cancer metastasis. Metastasis‐initiating oral cancer cells can exploit dietary lipids, including palmitic acid, to support metastatic progression [3, 4]. However, the effects of altered lipid metabolism depend on the lipid species and cellular context. Saturated fatty acids (SFAs) incorporated into phospholipids (PLs) can enhance oncogenic signaling and protect cancer cells from oxidative stress and chemotherapy, whereas changes in membrane saturation can also affect membrane fluidity, migration, and invasion [5, 6, 7, 8]. Importantly, several transcription factors coordinate these metabolic programs, including sterol regulatory element‐binding proteins (SREBFs) and peroxisome proliferator‐activated receptors (PPARs) [9].

PPAR‐gamma (PPARG) is a ligand‐activated nuclear receptor that regulates lipid metabolism, differentiation, inflammation, and metabolic homeostasis [10, 11]. Upon ligand binding, PPARG heterodimerizes with retinoid X receptors and regulates target genes through PPAR response elements. Its transcriptional activity depends on the cellular and metabolic context, including differentiation state, chromatin accessibility, and cofactor recruitment [12, 13]. Accordingly, PPARG has been associated with both tumor‐suppressive and tumor‐promoting effects [12, 14, 15]. This context dependence is particularly relevant when considering PPARG as a therapeutic target. PPARG agonists, including thiazolidinediones like pioglitazone and rosiglitazone, have shown anti‐proliferative, differentiation‐promoting, and chemopreventive effects in several experimental cancer models and have therefore attracted interest for cancer prevention and therapy [16, 17]. However, PPARG activation can also promote metastatic outgrowth in specific tumor and microenvironmental contexts [18]. In head and neck cancer, PPARG activation has been linked to differentiation and suppression of malignant phenotypes [16, 19], but increased PPARG expression and activity have also been associated with aggressive behavior and poor survival in head and neck squamous cell carcinoma (HNSCC) [20]. PPARG is furthermore among the nuclear receptors consistently downregulated in HNSCC compared with normal tissue, accompanied by alterations in lipid metabolic genes [21]. These divergent findings emphasize context dependence of PPARG function.

Lymphocyte cell‐specific protein tyrosine kinase (LCK) is a Src‐family non‐receptor tyrosine kinase best known for its role in T‐cell receptor (TCR) signaling. LCK associates with the cytoplasmic domains of the cluster of differentiation (CD) 4 and CD8 co‐receptors and phosphorylates the CD3/TCR complex, enabling recruitment and activation of zeta chain of TCR‐associated protein kinase 70 (ZAP70) and subsequent assembly of signaling complexes involving linker for activation of T cells (LAT) and SH2 domain‐containing leukocyte protein of 76 kDa (SLP‐76). These events initiate downstream pathways including calcium signaling, rat sarcoma (Ras) coupled with mitogen‐activated protein kinase (Ras/MAPK), and phosphatidylinositol 3‐kinase/protein kinase B (PI3K/AKT) signaling, which collectively regulate T‐cell activation, development, differentiation, and survival [22, 23, 24, 25]. Although primarily associated with lymphocytes, LCK expression and activity have also been detected in non‐hematopoietic cells and tumors, where LCK can function independently of the canonical TCR pathway and has been implicated in signaling processes regulating proliferation, survival, and cellular adaptation [22, 26].

We previously identified LCK as a driver of migration and invasion in OSCC using the heterogeneous SAS cell line [27]. Highly invasive subclones showed elevated LCK expression, whereas genetic or pharmacological LCK inhibition reduced invasion and promoted adherens junction formation and differentiation. Analysis of primary tumors and matched LNM further supported a role for LCK in invasive oral cancer cells [27]. These findings identified LCK as a potential therapeutic vulnerability but also raised the question of how a kinase classically associated with lymphocyte signaling promotes epithelial cancer cell invasion.

Here, we investigated downstream mechanisms linking LCK activity to metabolic adaptations supporting OSCC invasion. By integrating transcriptomic, epigenomic, and lipidomic analyses with genetic and pharmacological perturbation, we identified PPARG as a candidate downstream effector and examined its contribution to lipid composition, fatty acid metabolism, and invasion. We further investigated stearoyl‐CoA desaturase (SCD), a key regulator of the balance between SFAs and monounsaturated fatty acids (MUFAs), and its role in the invasive phenotype and sensitivity of PPARG‐activated cells to lipid peroxidation‐associated cell death.

## 2 Results

### 2.1 LCK Activity Upregulates PPARG Expression

Our previous study identified LCK as a driver of invasion in OSCC using highly and poorly invasive SAS subclones and patient‐derived tumor/metastasis pairs [27]. To identify pathways associated with their differential invasive behavior, highly invasive LCK‐high clone S and the poorly invasive LCK‐low clone F were embedded in Matrigel, extracted after 48 h, and subjected to RNA‐sequencing (RNA‐seq) (Figure 1A). We identified 323 differentially expressed genes (FDR ≤ 0.05; |log2(FC)| ≥ 1), including 212 upregulated and 111 downregulated in clone S (Table S1). Gene Ontology (GO) and Kyoto Encyclopedia of Genes and Genomes (KEGG) analyses revealed enrichment of the extracellular matrix and PPAR signaling pathways, respectively. Subsequent network analysis using the Search Tool for the Retrieval of Interacting Genes/Proteins (STRING) database identified PPARG as a central hub among upregulated genes (Figure 1B). Interestingly, analysis of our recently published OSCC cohort [28] revealed significantly higher PPARG expression in LNM^pos^ patients and in LNM compared with matched primary tumors, mirroring LCK expression (Figure 1C,D). Moreover, re‐analysis of previously generated datasets [27] showed reduced PPARG mRNA levels following LCK inhibition with A‐770041 (LCKi) or dasatinib, or following LCK knockdown by small interfering RNAs (siRNAs) (Figure 1E). PPARG protein abundance was likewise reduced upon LCK knockdown (Figure 1F) or pharmacological inhibition (Figure S1A).

**FIGURE 1:**
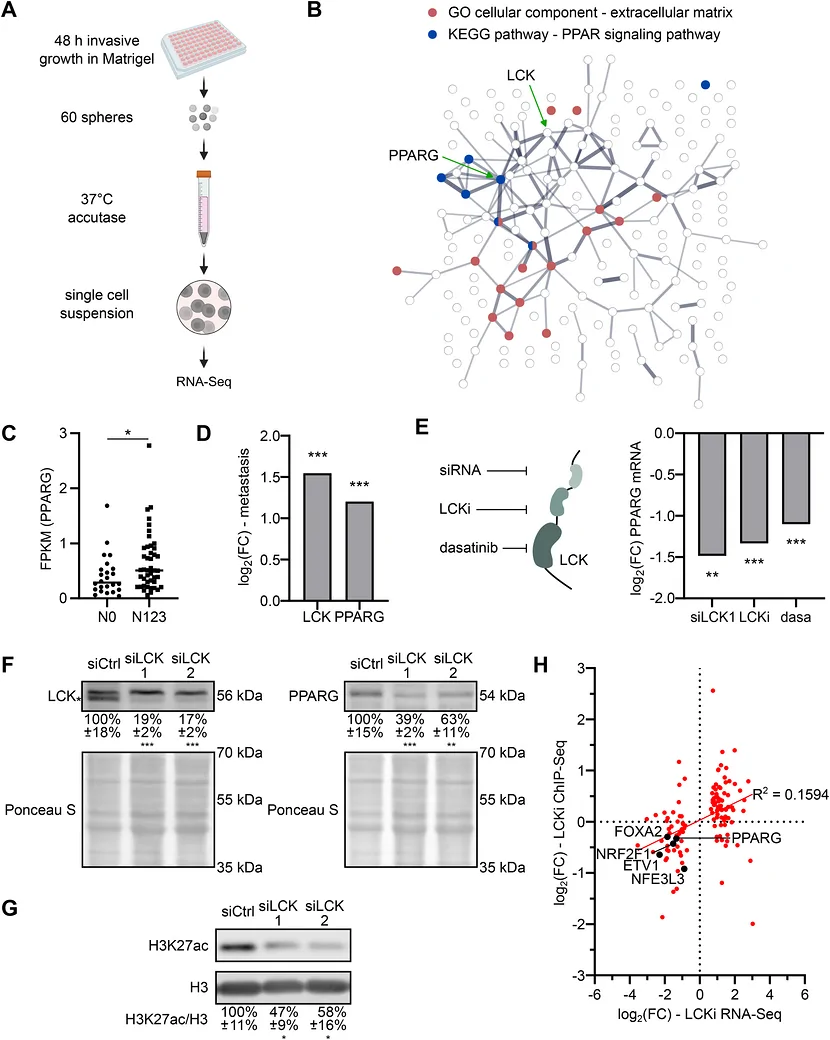
PPARG expression is regulated by LCK in SAS cells. (A) Schematic overview of sample preparation for gene expression analysis of cells grown in Matrigel. The scheme was created in BioRender. Gutschner, T. (2026) https://BioRender.com/bi00s1t. (B) STRING database network map of all significantly upregulated genes in highly invasive clone S extracted from Matrigel. Significantly overrepresented GO biological processes and KEGG pathways are highlighted. (C) PPARG transcript abundance (fragments per kilobase of transcript per million mapped reads, FPKM) in primary tumors with (N1–3) and without (N0) lymph node metastasis (LNM) (^*^ *p* ≤ 0.05, Mann–Whitney *U* test). (D) Relative LCK and PPARG mRNA expression in LNM compared to paired primary tumors (^***^FDR ≤ 0.001). Raw expression data for (C) and (D) taken from [28]. (E) PPARG mRNA expression after siRNA‐mediated LCK knockdown or inhibition via LCK inhibitor (LCKi, A‐770041) or dasatinib (dasa). RNA‐seq data from [27]. (^**^FDR ≤ 0.01, ^***^FDR ≤ 0.001). (F) Western blot to detect LCK, PPARG, and (G) global H3K27ac in SAS cells 48 h after siRNA‐mediated LCK knockdown. Ponceau S staining was used to control for equal loading. One‐way ANOVA was performed to compare to control siRNA (siCtrl, ^*^ *p* ≤ 0.05, ^**^ *p* ≤ 0.01, ^***^ *p* ≤ 0.001). (H) Correlation of H3K27ac peak enrichment (ChIP‐seq, *n* = 2) at selected genes after 24 h LCKi treatment versus gene expression (RNA‐seq) after 48 h LCKi treatment. Only differentially expressed genes with |Log_2_(FC) ≥ 1| and FDR ≤ 0.05 were considered for this analysis. Genes encoding oncogenic transcription factors showing a reduction of H3K27ac peak enrichment and decreased mRNA abundance upon LCK inhibition are highlighted.

Because LCK inhibition induces differentiation in oral cancer cells [27], we investigated whether altered chromatin regulation contributed to PPARG expression. Given the central role of histone H3 lysine 27 acetylation (H3K27ac) in marking active promoters and enhancers, we first examined whether LCK activity affects this transcription‐associated histone modification. Interestingly, we found that global H3K27ac levels were reduced following LCK knockdown or inhibition (Figure 1G and Figure S1B), and integration of H3K27ac chromatin immunoprecipitation‐sequencing (ChIP‐seq) and RNA‐seq showed reduced H3K27ac enrichment at the *PPARG* gene locus together with decreased *PPARG* transcript levels (Figure 1H). Similar changes were observed for *forkhead box A2* (*FOXA2*), *nuclear receptor subfamily 2 group F member 1* (*NR2F1*), *ETS variant transcription factor 1* (*ETV1*), and *NFE2‐like BZIP transcription factor 3* (*NFE2L3*) (Table S2).

To further characterize the relationship between LCK and chromatin regulation, we examined the subcellular localization of endogenous LCK by biochemical fractionation. LCK was detected in both cytoplasmic and nuclear fractions (CF and NF), with GAPDH and PARP confirming fraction purity (Figure S1C). LCK‐specific signals were reduced following LCK knockdown, confirming the specificity of the detected protein. In order to determine whether altered H3K27ac reflected changes in chromatin‐modifying enzymes, we analyzed transcriptomic datasets following genetic and pharmacological LCK inhibition. Expression of examined histone acetyltransferases, deacetylases, sirtuins, acetyl‐CoA‐generating enzymes, or other chromatin regulators was not consistently altered. In particular, *EP300* mRNA changed only minimally following LCK knockdown (log2(FC) = 0.154) or pharmacological inhibition (log2(FC) = 0.118), and p300 protein abundance was unchanged after LCK depletion (Figure S1D).

Together, these findings identify PPARG as a downstream component associated with LCK‐driven invasion and implicate LCK in the regulation of H3K27 acetylation.

### 2.2 PPARG Induces Invasiveness Downstream of LCK

We next examined the effects of PPARG modulation on three‐dimensional (3D) growth and invasion. LCK inhibition markedly reduced SAS invasion without significantly affecting spheroid growth. Similarly, PPARG inhibition with GW9662 (GW) reduced invasion of parental SAS cells and motility of highly invasive clone S, whereas PPARG activation with rosiglitazone (Rosi) strongly enhanced invasion. The pro‐invasive effect of rosiglitazone persisted during LCK inhibition, although both PPARG activation and inhibition impaired spheroid growth (Figure 2A–C).

**FIGURE 2:**
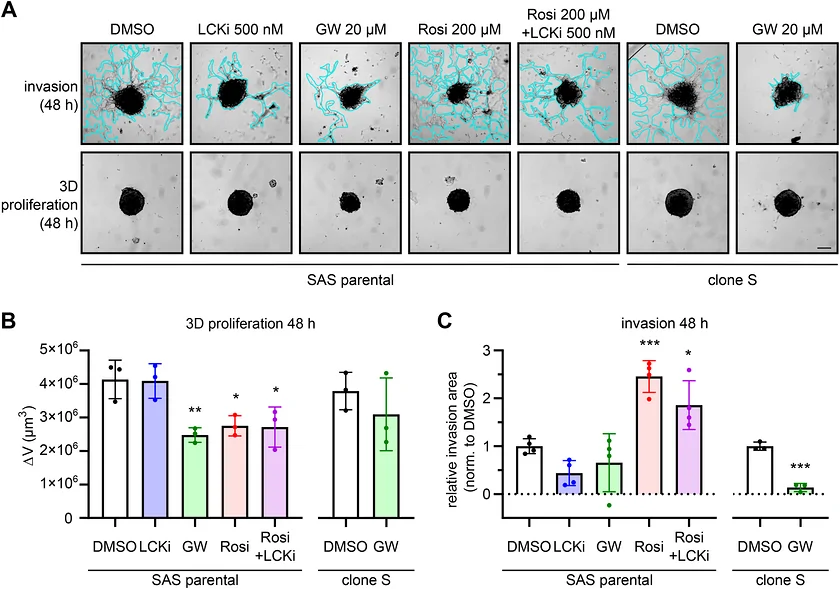
Modulation of PPARG activity affects SAS spheroid growth and invasion. (A) Representative pictures of SAS spheroids embedded in Matrigel (upper row) or grown in normal media (lower row) for analysis of (B) 3D proliferation or (C) invasion. Cells were treated for 48 h with indicated amounts of LCK inhibitor (LCKi/A‐770041) and/or PPARG agonist (rosiglitazone, Rosi), or PPARG antagonist (GW9662, GW). One‐way ANOVA was performed to compare experimental samples to DMSO treatment (*n* = 3; ^*^ *p* ≤ 0.05, ^**^ *p* ≤ 0.01, ^***^ *p *≤ 0.001; Scale bar = 200 µm).

We further examined these effects in UPCI‐SSC‐084 (oral cancer), Chl‐1 (melanoma) as well as in the pancreatic cancer‐derived cell lines PaTu8902 and PaTu8988T (Figure S2). LCK inhibition reduced invasion in all four cell lines, whereas rosiglitazone produced heterogeneous effects: invasion was unchanged in Chl‐1, increased in PaTu8988T, reduced in PaTu8902, and transiently increased in UPCI‐SSC‐084 cells before modestly decreasing at later time points. LCK inhibition reduced growth in PaTu8988T and Chl‐1 cells, while rosiglitazone modestly impaired growth in SAS and PaTu8988T cells. Thus, PPARG activation promotes invasiveness downstream of LCK in SAS cells, whereas its effects on motility and growth depend on cellular context.

### 2.3 Lipid Storage Capacity Correlates With LCK and PPARG Activity and Cell Motility

To investigate the relationship between LCK/PPARG signaling, lipid homeostasis, and invasion, we analyzed lipid metabolism in a panel of previously established high‐ and low‐invasive SAS clones [27]. Following treatment with 125 µM oleic acid, highly invasive clones showed weaker BODIPY 558/568 C12 fluorescence, indicating differences in lipid droplet formation (Figure 3A,B). In SAS cells, both LCK and PPARG inhibition increased lipid droplet formation, whereas rosiglitazone reduced the BODIPY fluorescence (Figure 3C,D). Combined LCK inhibition and rosiglitazone treatment did not restore the lipid droplet phenotype. Thus, LCK and PPARG activities are associated with lipid storage capacity in SAS cells, with highly invasive cells displaying reduced lipid droplet‐associated BODIPY fluorescence.

**FIGURE 3:**
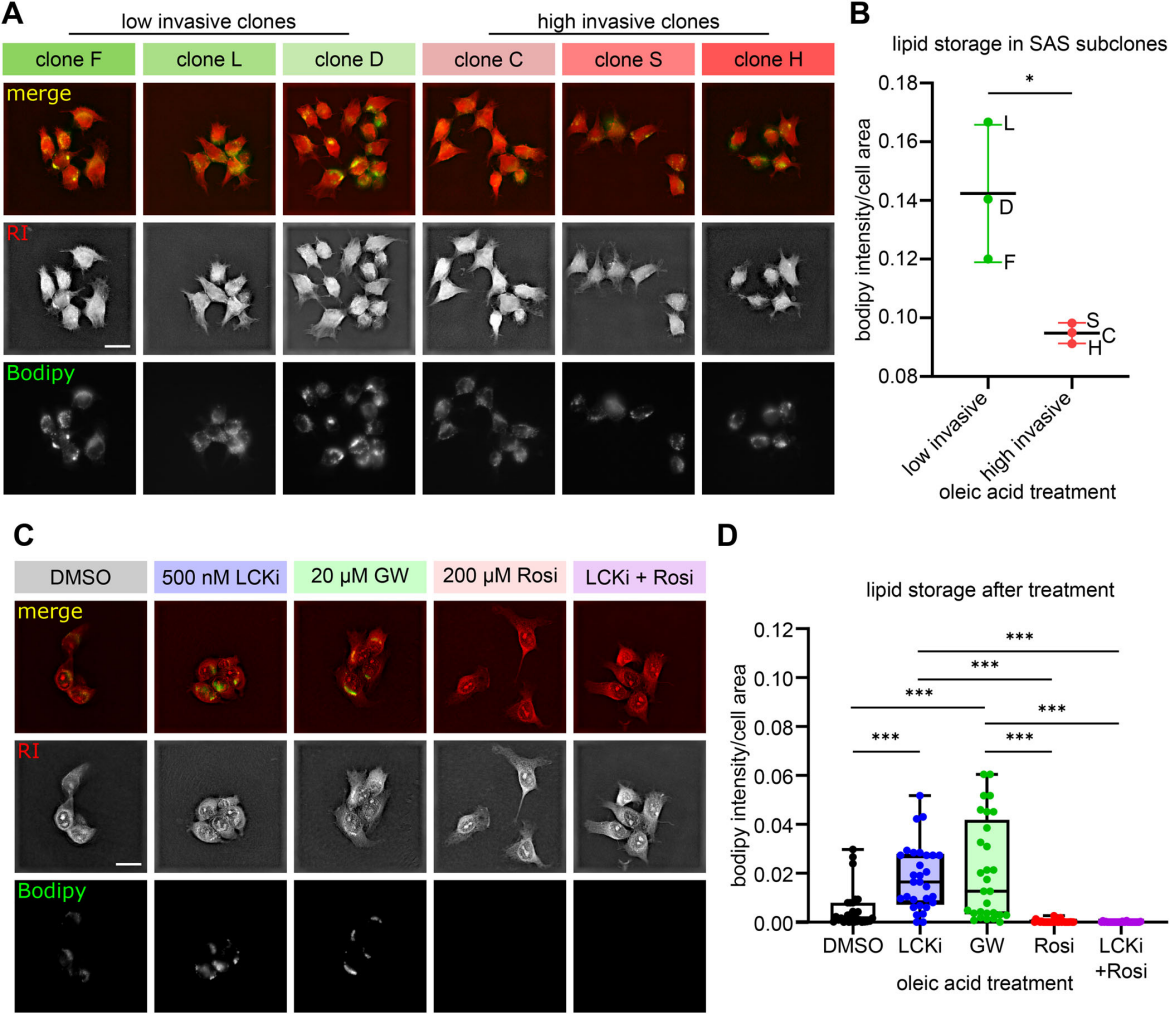
Amount of lipid droplets differs between high and weak invasive SAS clones and increases upon LCK and PPARG inhibition. (A) Representative pictures of high and low invasive clones treated with 125 µM oleic acid labeled with BODIPY 558/568 C12. Refraction index (RI) and BODIPY 558/568 C12 fluorescence are shown. Scale bar = 20 µM. (B) Quantification of BODIPY 558/568 C12 intensity in individual clones grouped according to their invasive capacities. Student's *t*‐test was used to compare both groups (*n* = 3; ^*^ *p* ≤ 0.05). (C) Representative pictures showing RI and BODIPY 558/568 C12 fluorescence of parental SAS cells treated with 125 µM oleic acid and LCK inhibitor (LCKi), PPARG inhibitor (GW), or PPARG activator (Rosi). Scale bar = 20 µm. (D) Quantification of BODIPY 558/568 C12 intensity in treatment groups. Individual data points represent analyzed pictures. One‐way ANOVA was performed to compare different treatments to DMSO (^*^ *p* ≤ 0.05, ^***^ *p *≤ 0.001).

### 2.4 Phospholipid Composition Is Altered by LCK/PPARG Signaling

To characterize the lipid composition associated with the invasive phenotype, we performed untargeted lipidomics of high‐ and low‐invasive SAS clones. We identified 39 significantly upregulated features (*p* ≤ 0.05, |log2(FC)| ≥ 1), including 30 PLs containing C16 and/or C18 fatty acid residues. In contrast, free fatty acids (FFAs) containing C16 or C18 chains, and two C16/C18‐containing fatty acyl esters of hydroxy fatty acids (FAHFAs) were significantly reduced in highly invasive clones (Figure S3A).

We next analyzed lipidomic changes following LCK depletion and PPARG modulation. PLs containing at least one C16 or C18 residue, including saturated and unsaturated species, increased following LCK knockdown, PPARG inhibition, and PPARG activation, with the highest levels observed in rosiglitazone‐treated cells. Notably, rosiglitazone‐induced upregulation was retained in LCK‐deficient cells (Figure 4A). In addition, LCK knockdown and PPARG modulation increased the levels of PLs with at least one C20 or C22 fatty acid residue, although rosiglitazone reduced these species in LCK‐depleted cells (Figure 4B).

**FIGURE 4:**
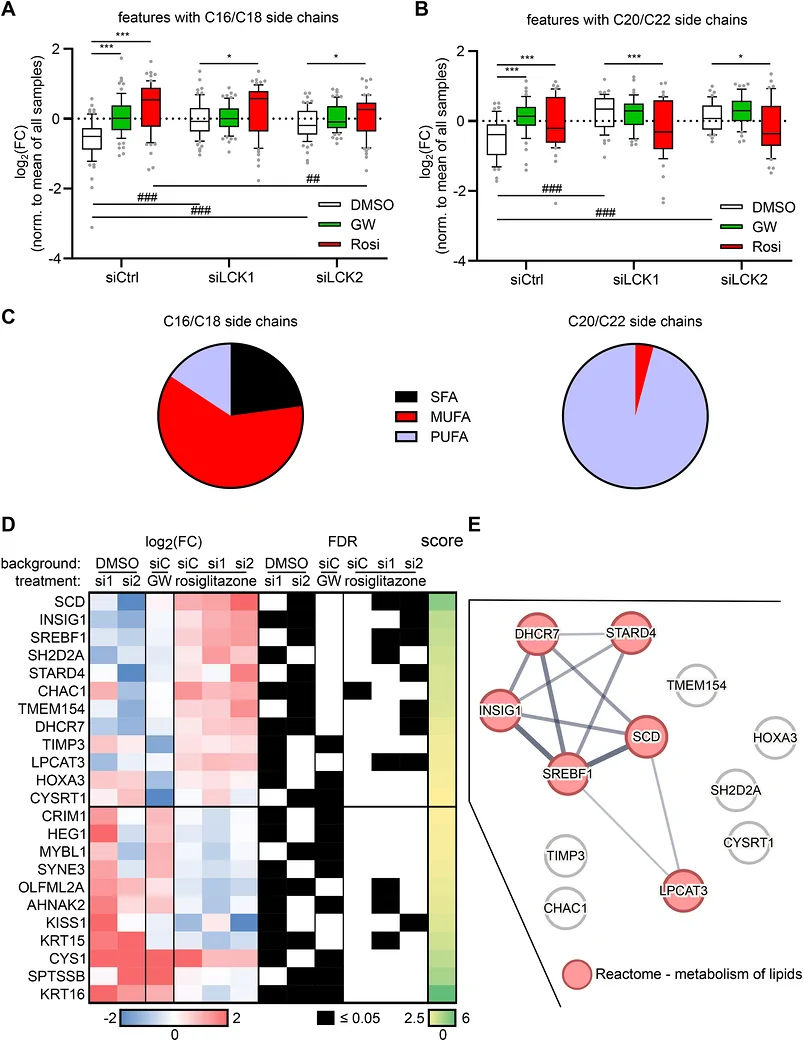
LCK/PPARG signaling controls phospholipid composition and expression of lipid metabolism‐associated genes. Untargeted lipidomics identified multiple significantly deregulated features in LCK‐depleted cells alone and/or in combination with PPARG inhibition or activation. (A) Features with C16 or C18 side chains, and (B) features with C20 or C22 side chains were identified by MS/MS analysis. Two‐way ANOVA was performed to compare samples with siCtrl (^##^ *p* ≤ 0.01, ^###^ *p *≤ 0.001) or DMSO control treatment (^*^ *p* ≤ 0.05, ^***^ *p *≤ 0.001). (C) Saturation status of these side chains is shown: saturated fatty acid (SFA), monounsaturated fatty acid (MUFA), and polyunsaturated fatty acid (PUFA). (D) Heatmap visualizing significant expression changes of protein‐coding genes based on RNA‐seq analyses performed in SAS cells deprived of LCK and/or treated with GW9662 or rosiglitazone. A LCK/PPARG correlation score was used to identify genes that are regulated in same direction upon LCK‐depletion or PPARG inhibition, yet in the opposite direction compared to PPARG activation with and without parallel LCK knockdown. Genes were filtered for LCK/PPARG signaling score ≥ 2.5, as well as statistical significance only including genes with FDR ≤ 0.05 after LCK siRNA treatment and a significant expression change (FDR ≤ 0.05) upon modulation of PPARG activity. (E) Network map from STRING database showing genes that positively correlated with LCK/PPARG signaling. The significantly overrepresented Reactome gene set “Metabolism of lipids” is highlighted.

Longer‐chain fatty acids, such as C20 and C22, are more likely to be polyunsaturated fatty acids (PUFAs), whereas shorter chains like C16 and C18 are typically MUFAs, with some being SFAs. Indeed, C16/C18 side chains consisted predominantly of MUFAs (61.4%), followed by SFAs (22.9%) and PUFAs (15.7%), whereas C20/C22 side chains were predominately PUFAs (95.9%) (Figure 4C). Correspondingly, MUFA‐ and PUFA‐containing PLs showed response patterns similar to C16/C18‐ and C20/C22‐containing species, respectively, while PLs containing only SFAs changed minimally (Figure S3B–D).

These data demonstrate that LCK depletion and PPARG modulation reshape PL composition, with PPARG activation partially reversing the lipidomic changes associated with LCK depletion.

### 2.5 Lipid Metabolism‐Associated Genes Are Regulated by LCK/PPARG Signaling

To identify genes associated with the lipidomic changes, we performed RNA‐seq following LCK knockdown alone or combined with PPARG activation or inhibition. We integrated gene expression changes across LCK‐depleted, PPARG‐inhibited, and PPARG‐activated SAS cells, in both, control and LCK‐depleted backgrounds to derive an LCK/PPARG signaling score. Applying a score threshold of ≥ 2.5 and requiring significant differential expression (FDR ≤ 0.05) in at least one comparison identified 12 positively and 11 negatively associated genes (Figure 4D and Table S3). *KRT15* and *KRT16* were among the most strongly negatively associated genes, whereas the positively associated genes were significantly enriched for the Reactome pathway “Metabolism of lipids” (Figure 4E). *SCD*, *INSIG1* (*insulin‐induced gene 1*), and *SREBF1* (*sterol regulatory element‐binding transcription factor 1*) ranked among the top candidate genes and showed strong co‐expression in the STRING analysis.

Collectively, these data identify a set of genes associated with LCK/PPARG signaling and highlight lipid metabolism as a major enriched pathway, with *SCD*, *INSIG1*, and *SREBF1* emerging as candidate downstream effectors.

### 2.6 Fatty Acid Synthesis‐Associated Genes Promote Cell Proliferation and Invasion

We next examined the functional relevance of SREBF1 and its downstream fatty acid synthesis‐associated genes using inhibitors of SREBF1 (fatostatin [29]), ACC (TOFA [30]), FASN (cerulenin [31]), and SCD (CAY10566 [32]) (Figure 5A). Fatostatin, TOFA, and cerulenin reduced spheroid growth in a concentration‐dependent manner, whereas CAY10566 reduced growth by approximately 50% at 1 µM, with no further reduction at higher concentrations (Figure 5B). A low dose of rosiglitazone (50 µM) had no effect on growth alone and did not substantially alter the effects of fatostatin or cerulenin. In contrast, rosiglitazone sensitized cells to TOFA and CAY10566; at 10 µM TOFA or 1 µM CAY10566, co‐treatment caused spheroid disintegration, preventing reliable quantification of spheroid size (Figure 5C).

**FIGURE 5:**
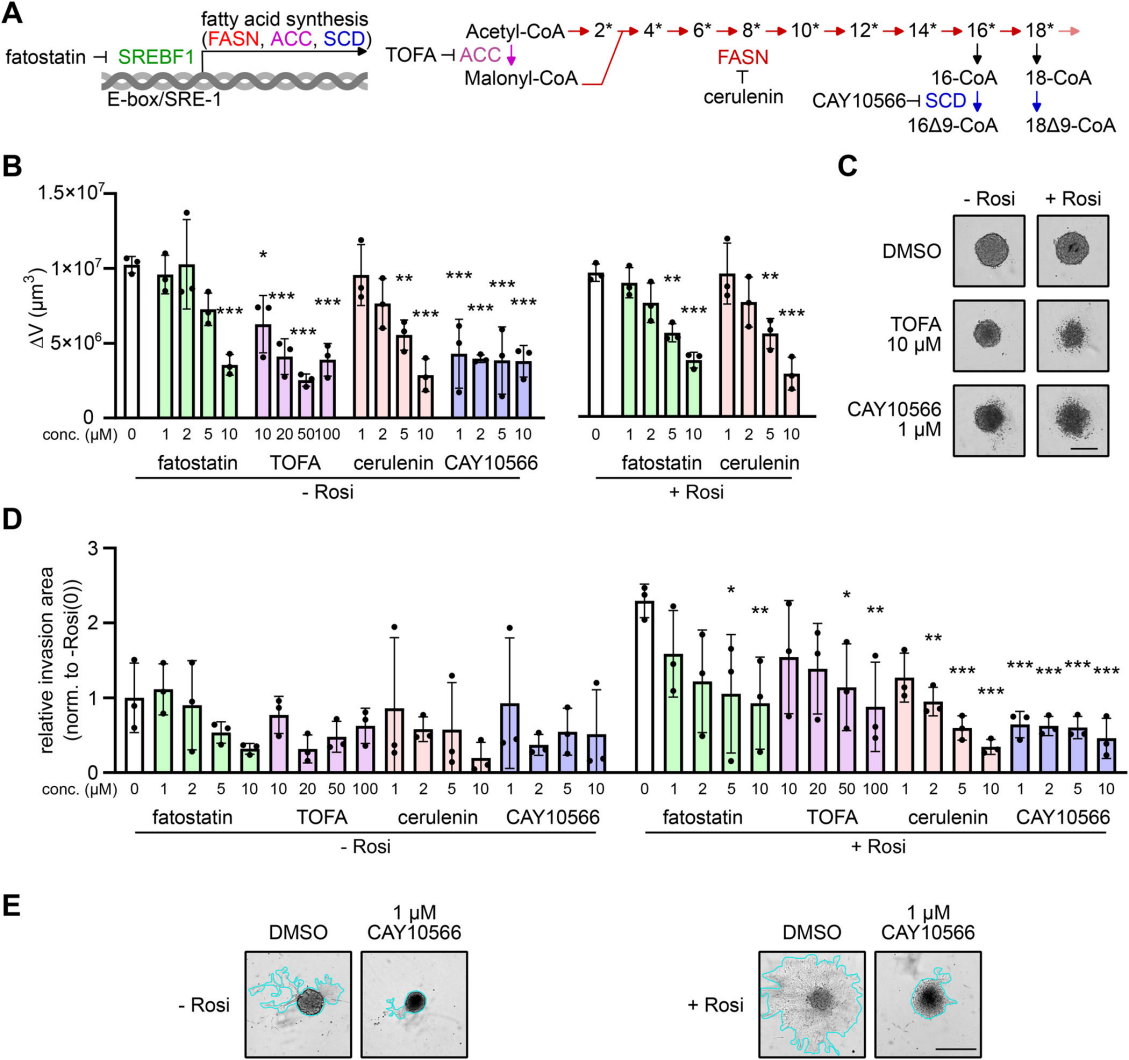
Inhibition of fatty acid synthesis‐associated genes impairs proliferation and invasion. (A) Regulators of fatty acid synthesis, namely SREBF1, ACC, FASN, and SCD, can be targeted by fatostatin, TOFA, cerulenin, and CAY10566, respectively. (B) Spheroid growth of SAS treated for 24 h with increasing amounts of the respective inhibitors alone or in combination with 50 µM rosiglitazone. One‐way ANOVA was performed to compare different treatments to DMSO (*n* = 3, ^*^ *p* ≤ 0.05, ^**^ *p* ≤ 0.01, ^***^ *p *≤ 0.001). (C) Representative pictures of spheroids treated with single agents (TOFA or CAY10566) and in combination with 50 µM rosiglitazone. Scale bar = 400 µm. (D) Matrigel‐based three‐dimensional invasion of SAS cells treated for 24 h with increasing amounts of small molecule inhibitors alone or in combination with 50 µM rosiglitazone. Two‐way ANOVA was performed to compare different treatments to DMSO (*n* = 3, ^*^ *p* ≤ 0.05, ^**^ *p* ≤ 0.01, ^***^ *p *≤ 0.001). (E) Representative pictures of Matrigel invasion after treatment of SAS cells with 1 µM CAY10566 for 24 h. Scale bar = 800 µm.

In Matrigel invasion assays, the inhibitors produced variable responses without significant dose‐dependent effects in the absence of rosiglitazone. Nevertheless, 10 µM fatostatin, 20 µM TOFA, 10 µM cerulenin, and 2 µM CAY10566 reduced motility by approximately 70%–80% (Figure 5D). Rosiglitazone increased invasion more than twofold, and this effect was attenuated dose‐dependently by fatostatin, TOFA, and cerulenin. CAY10566 was particularly effective, with 1 µM suppressing invasion both with and without PPARG activation (Figure 5E).

These findings identify fatty acid synthesis‐associated pathways as regulators of SAS cell growth and invasion and highlight SCD inhibition as an effective strategy to suppress PPARG‐driven invasion.

### 2.7 PPARG Activation Creates a Dependency on SCD to Maintain Cell Viability

We next investigated the relationship between PPARG activation and SCD activity in additional cancer cell lines. Rosiglitazone consistently increased SCD mRNA expression more than twofold in SAS, UPCI‐SSC‐084, PaTu8988T, and PaTu8902 cells (Figure S4A). Low‐dose CAY10566 (0.01, 0.1, and 1 µM) had little effect on viability after 48 h, whereas combined CAY10566 and rosiglitazone treatment significantly reduced cell viability, particularly in SAS and PaTu8902 cells (Figure 6A). Consistent with increased cell death, 0.1 µM CAY10566 produced a modest increase in PARP cleavage after 24 h (1.42‐fold ± 0.61), which increased approximately fourfold upon rosiglitazone co‐treatment (Figure 6B). After 48 h, PARP cleavage increased more than 40‐fold following CAY10566 treatment alone, with a slight additive effect of rosiglitazone (Figure 6C). Because SCD inhibition has been associated with lipid peroxidation, we next evaluated this lipid damage using BODIPY 581/591 C11, and we observed a dose‐dependent increase of BODIPY 581/591 C11 oxidation in CAY10566‐treated cells (Figure 6D). Thus, PPARG activation increases SCD expression and sensitizes cancer cells to SCD inhibition, accompanied by increased PARP cleavage and lipid peroxidation.

**FIGURE 6:**
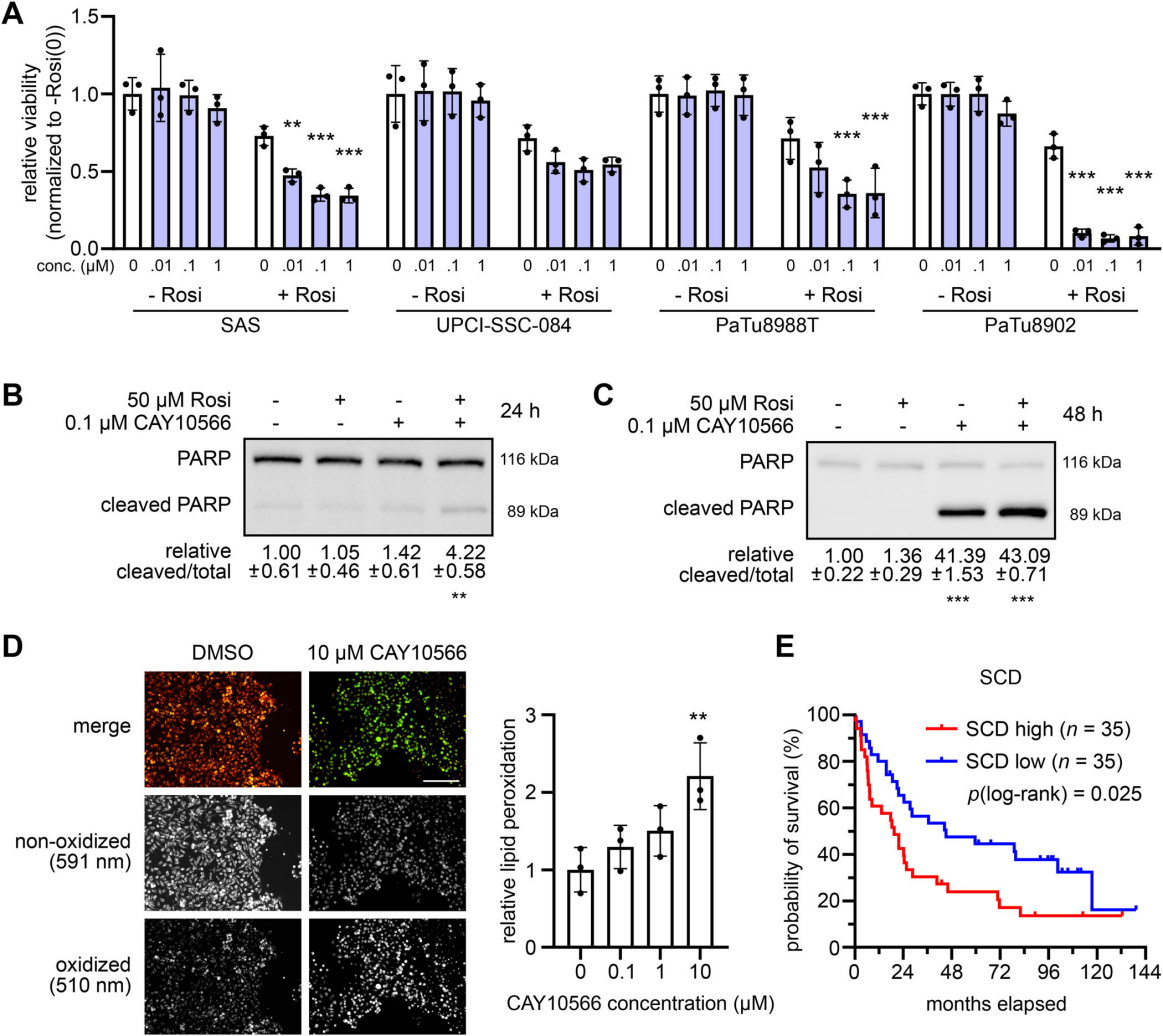
SCD is a biomarker and therapeutic target in OSCC. (A) Viability assay (MTT) after treatment of attached cells with different CAY10566 concentrations alone or in combination with 50 µM rosiglitazone for 48 h. Two‐way ANOVA was performed to compare treated samples to DMSO control (*n* = 3, ^**^ *p* ≤ 0.01, ^***^ *p* ≤ 0.001). (B) Western blot analysis showing expression of full‐length and cleaved PARP1 in SAS cells treated with 50 µM rosiglitazone alone or in combination with 0.1 µM CAY10566 for 24 h, or (C) treated for 48 h in 2D culture. Two‐way ANOVA was performed to compare to DMSO (*n* = 3, ^**^ *p* ≤ 0.01, ^***^ *p* ≤ 0.001). (D) Analysis of lipid peroxidation in SAS cells after SCD inhibition for 3 h using BODIPY 581/591 C11. Scale bar = 200 µm. One‐way ANOVA was performed to compare to DMSO (*n* = 3, ^**^ *p* ≤ 0.01). (E) Kaplan–Meier curves for overall survival of OSCC patients [28] stratified by SCD mRNA expression. Samples (*n* = 70) were divided by median separation.

### 2.8 High Expression of SCD Is Associated With Poor Survival in Head and Neck Cancers

To assess the clinical relevance of SCD expression, we analyzed publicly available transcriptomic data from The Cancer Genome Atlas (TCGA) HNSCC cohort using GEPIA2 portal [33]. SCD expression was increased in primary tumors compared with normal tissues, particularly in the basal and classical subtypes (Figure S4B,C). Moreover, in our previously published OSCC patient cohort [28], high SCD expression was significantly associated with poor OS (Figure 6E). Elevated *SCD* transcript levels were also significantly associated with poor disease‐free survival (DFS) in atypical and basal subtype samples within the TCGA HNSCC cohort, with a similar trend in the classical subtype (Figure S4D). These findings associate elevated SCD expression with HNSCC and adverse clinical outcome.

## 3 Discussion

Oral cancer frequently progresses through local invasion and cervical LNM, which remain major determinants of patient outcome. We previously identified LCK as a driver of invasion and migration, suggesting a tumor‐cell‐intrinsic role for LCK in OSCC progression [27]. Because LCK is also essential for TCR signaling [22], direct systemic inhibition may present therapeutic challenges. The present study therefore sought to define downstream mechanisms connecting LCK activity to the invasive phenotype and potentially providing more selective therapeutic opportunities. Our data identify PPARG as a functional downstream effector of LCK and link this pathway to H3K27 acetylation, lipid remodeling, and SCD‐dependent metabolic adaptation. In the LCK‐high SAS model, PPARG activation promoted invasion and increased SCD expression, whereas SCD inhibition reduced invasion and viability and increased lipid peroxidation. These findings establish a connection between LCK signaling, PPARG‐dependent lipid metabolism, and invasive behavior in OSCC.

Our data suggest that LCK regulates PPARG at a transcriptional and chromatin‐associated level. LCK inhibition reduced both PPARG expression and global H3K27ac, while H3K27ac enrichment at the *PPARG* locus decreased together with *PPARG* transcript levels. H3K27ac marks active promoters and enhancers and is therefore consistent with transcriptional regulation of *PPARG* [34, 35, 36]. Detection of endogenous LCK in both cytoplasmic and nuclear compartments provides a potential spatial basis for this association and is consistent with previous evidence for nuclear LCK and its association with chromatin [37]. Importantly, our additional analyses argue against a simple mechanism in which LCK controls H3K27ac by altering the expression or abundance of major H3K27ac‐modifying enzymes. Expression of examined acetyltransferases, deacetylases, sirtuins, and other relevant regulators was not consistently affected by LCK inhibition, and p300 protein abundance remained unchanged. Reduced H3K27ac is therefore unlikely to reflect reduced abundance of these enzymes. Nuclear LCK could instead influence chromatin through regulation of enzyme activity, chromatin recruitment, subcellular localization, or phosphorylation of chromatin‐associated proteins. These mechanisms remain to be established. Our data support a role for LCK in regulating H3K27ac‐associated transcription but do not identify the molecular intermediates.

The effects of PPARG activation further illustrate its context dependence in cancer. PPARG regulates differentiation, lipid metabolism, and immune regulation, and its transcriptional output depends on the cellular state, chromatin accessibility, and cofactor recruitment [12, 13]. Consequently, both tumor‐suppressive and tumor‐promoting activities have been described [12, 14, 15]. In head and neck cancer, PPARG agonists have shown anti‐proliferative, differentiation‐promoting, and chemopreventive effects [16, 19, 38], whereas PPARG can promote tumor progression in defined contexts [18].

In our experiments, PPARG activation promoted invasion in SAS cells and remained pro‐invasive despite LCK inhibition, supporting PPARG as a downstream functional mediator of LCK‐dependent invasion. However, rosiglitazone produced heterogeneous effects across additional cancer cell lines, emphasizing the importance of cellular context. Moreover, thiazolidinediones like rosiglitazone can exert PPARG‐independent effects [39, 40, 41]. Our findings therefore do not support a universal pro‐invasive role for PPARG in squamous carcinomas but suggest that PPARG can function as a pro‐invasive LCK effector in a defined subset of OSCC cells.

The connection between LCK/PPARG signaling and lipid metabolism provides a potential explanation for this context‐dependent phenotype. Lipid metabolic reprogramming contributes to membrane synthesis, signaling, energy production, stress adaptation, and metastatic dissemination, and altered fatty acid metabolism has been associated with HNSCC progression and therapeutic resistance [42]. Our lipidomic analyses extend these observations by showing that LCK depletion and PPARG modulation alter PL composition, particularly species containing distinct C16/C18 and C20/C22 fatty‐acyl chains. Thus, LCK/PPARG signaling does not simply alter total cellular lipid abundance but reshapes lipid composition in OSCC cells.

Our transcriptional analyses provide a mechanistic framework for these changes. Genes associated with LCK/PPARG signaling were enriched for lipid metabolic functions, with *SREBF1*, *INSIG1*, and *SCD* emerging as prominent candidates. Inhibition of SREBF1 and its downstream lipogenic enzymes ACC, FASN or SCD impaired spheroid growth or invasion. These findings are consistent with the established contribution of de novo lipogenesis to malignant growth and invasion. SCD is of particular interest because it converts SFAs into MUFAs and thereby directly influences membrane lipid composition. Increased SCD activity promotes tumor growth and survival in several cancer types, while SCD inhibition has shown antitumor effects in preclinical models [43, 44]. Previous studies have also reported increased SCD expression in HNSCC and reduced proliferation and invasion following SCD inhibition [45, 46]. Our data extend these findings by placing SCD within an LCK/PPARG signaling framework and suggest that this metabolic program contributes to the invasive phenotype.

The relationship between SCD, MUFAs, PUFAs, and lipid peroxidation provides a further mechanistic dimension. SCD‐generated MUFAs influence membrane composition and reduce the availability of highly oxidizable PUFA‐containing PLs. Experimental studies have shown that MUFAs can suppress ferroptosis by reducing the incorporation of oxidizable PUFAs into membrane PLs, whereas SCD activity can protect cancer cells against ferroptotic death [47, 48]. In our model, PPARG activation increased SCD expression and altered the lipidomic phenotype, whereas SCD inhibition increased lipid peroxidation and reduced viability. These observations suggest that PPARG‐driven SCD activity shifts lipid metabolism toward a more MUFA‐rich state that limits lipid peroxidation and supports survival during invasive growth. However, our data demonstrate increased lipid peroxidation following SCD inhibition but do not establish ferroptosis as the sole mechanism of cell death. Direct demonstration of ferroptotic death will be required to define this mechanism more precisely.

Importantly, the finding that PPARG activation increases dependence on SCD also has potential therapeutic implications. SCD has emerged as a metabolic vulnerability in several tumor types, and its pharmacological inhibition can impair tumor growth in vitro and in vivo [44, 49, 50]. Our results indicate that this vulnerability may be particularly relevant in PPARG‐active, LCK‐high OSCC cells. Thus, rather than considering PPARG activation itself as a therapeutic endpoint, the LCK/PPARG/SCD axis may define a metabolic state that identifies tumors susceptible to downstream metabolic intervention.

These findings are relevant to the previous interest in PPARG agonists as cancer‐preventive or therapeutic agents. Anti‐proliferative and differentiation‐promoting effects of PPARG agonists have been reported in oral cancer models [16, 19], and our findings do not contradict these observations. Instead, they suggest that PPARG activation can have different consequences in premalignant and established tumor cells or across distinct tumor‐cell states. PPARG may also exert pro‐tumorigenic effects outside the malignant cell compartment through stromal and inflammatory responses [51, 52, 53]. These findings reinforce the need to consider both tumor‐cell‐intrinsic and microenvironmental effects when evaluating PPARG‐directed therapies. Moreover, the possibility that PPARG activation enhances invasion in a subset of LCK‐high tumors supports careful assessment of tumor‐cell‐intrinsic PPARG activity and biomarker‐based patient stratification.

The association of SCD expression with poor outcome in our OSCC cohort and selected TCGA HNSCC subtypes further supports the clinical relevance of this metabolic program. These associations remain correlative, however. LCK presents an additional challenge in clinical datasets because it is abundantly expressed by infiltrating lymphocytes, which can obscure tumor‐cell‐intrinsic LCK expression in bulk transcriptomic or conventional immunohistochemical analyses. This distinction is particularly important in HNSCC, where immune infiltration itself can have strong prognostic associations. Our previous work demonstrated that the relationship between LCK expression and clinical outcome differs between tumor subtypes, supporting the concept that tumor‐cell‐intrinsic LCK biology may be distinct from prognostic signals generated by immune‐cell infiltration [27]. Future studies should therefore assess LCK, PPARG, and SCD specifically in malignant cells, ideally using spatial or multiplex approaches, to determine whether co‐activation of this pathway defines a clinically relevant OSCC subgroup.

### 3.1 Limitations of the Study

Several limitations should be considered when interpreting our findings. First, the mechanistic experiments were predominantly performed in established cancer cell models, with SAS cells providing the most consistent evidence for the LCK/PPARG/SCD axis. Although additional cell lines confirmed several pathway components, the heterogeneous response to PPARG activation indicates that its effects are context‐dependent. Second, the study relies substantially on pharmacological modulation of LCK, PPARG, and SCD, and target‐independent effects cannot be excluded. Genetic LCK depletion and multiple independent experimental readouts addressed this limitation, but genetic perturbation of PPARG and SCD would provide additional mechanistic validation. Third, although our findings implicate LCK in H3K27ac regulation and identify nuclear LCK localization, the molecular mechanism linking LCK to chromatin remains unresolved. Similarly, increased lipid peroxidation following SCD inhibition is consistent with ferroptosis‐associated cell death but does not establish ferroptosis as the underlying mechanism. Finally, the complete LCK/PPARG/SCD axis has not yet been validated in vivo, particularly with respect to OSCC invasion, LNM, and therapeutic response. Clinical analyses are likewise correlative and do not establish co‐activation of the pathway within malignant cells. Orthotopic models and tumor‐cell‐resolved spatial or single‐cell analyses will therefore be important to define the translational relevance of this pathway.

In summary, our findings identify an LCK/PPARG/SCD axis linking signaling and chromatin regulation to lipid remodeling and invasive behavior in OSCC. This pathway may represent a context‐dependent metabolic vulnerability and warrants further investigation as a potential therapeutic target.

## 4 Materials and Methods

### 4.1 Cell Culture and Drug Treatments

Human OSCC cell lines SAS (kind gift from Prof. Dr. Daniel Zips) and UPCI‐SSC‐084 (ACC 878, DSMZ, Braunschweig, Germany) were cultured in RPMI‐1640 supplemented with 10% fetal bovine serum (FBS), 100 units/mL penicillin, 100 µg/mL streptomycin, 2 mmol/L L‐glutamine (all Thermo Fisher Scientific, Waltham, MA, USA), and 1 mmol/L sodium pyruvate (Sigma Life Science, St. Louis, MO, USA). The human cancer cell lines Chl‐1 (ATCC CRL‐3619, Manassas, VA, USA), PaTu8988T, and PaTu8902 (ACC 162 and ACC 179, DSMZ) were cultured in DMEM (Thermo Fisher Scientific) supplemented with 10% FBS. All cell lines were maintained at 37°C and 5% CO_2_ and regularly tested for mycoplasma contamination. Cells were treated with DMSO (Carl Roth, Karlsruhe, Germany), A‐770041 (Axon Medchem, Reston, VA, USA), rosiglitazone and GW9662 (Sigma‐Aldrich, St. Louis, MO, USA), or cerulenin, fatostatin, CAY10566, and TOFA (Targetmol, Boston, MA, USA), as indicated.

### 4.2 Matrigel Extraction

High‐invasive SAS clone S‐eGFP or low‐invasive SAS clone F‐mCherry cells [27] (6 × 10^4^) were seeded each into 60 wells of an ultra‐low attachment plate (Corning, Corning, NY, USA) each. After 24 h, spheres were embedded in Matrigel (Corning) and incubated for an additional 48 h. The 60 spheres per clone, including surrounding Matrigel, were collected, centrifuged at 1500 × g for 5 min, and incubated in 4 mL Accutase (Biowest, Nuaillé, France) at 37°C for 15 min. Centrifugation and Accutase treatment were repeated until spheroids were dissociated and Matrigel removed. The resulting single‐cell suspensions were used for RNA isolation.

### 4.3 RNA Isolation and Reverse Transcription‐Quantitative Polymerase Chain Reaction (RT‐qPCR)

Total RNA was isolated by acid guanidinium thiocyanate‐phenol‐chloroform extraction as described previously [54, 55], and resuspended in ultra‐pure water (Life Technologies, Carlsbad, CA, USA). Two micrograms of RNA per sample were reverse‐transcribed using the M‐MLV Reverse Transcriptase system (Promega, Madison, WI, USA). qPCR was performed using PrimaQuant CYBR‐qPCR Mastermix (Steinbrenner Laborsysteme, Wiesenbach, Germany) on a Light Cycler I (Roche, Basel, Switzerland).

SCD expression was measured using SCD_forward (TTCTACACCTGGCTTTGGGG) and SCD_reverse (GAAAACTTGTGGTGGGCACG) primers. RPLP0 (forward: GGCGACCTGGAAGTCCAACT; reverse: CCATCAGCACCACAGCCTTC) and PPIA (forward: GTCAACCCCACCGTGTTCTT; reverse: CTGCTGTCTTTGGGACCTTGT) served as reference genes.

### 4.4 RNA‐Sequencing, Pathway Analysis, and Signaling Score Calculations

Two micrograms of RNA were used for library preparation and sequencing by Genewiz (Leipzig, Germany). Adapter sequences and low‐quality read ends were removed using Cutadapt [56]. Reads were aligned to the human reference genome (UCSC hg38) using HiSat2 [57], and primary alignments were extracted and BAM files indexed using Samtools [58]. FeatureCounts [59] was used for gene‐level read summarization based on ENSEMBL GRCmh38 v110 annotation [60]. Differential gene expression was determined with edgeR v4.2.2 using trimmed mean of M‐values (TMM) normalization [61, 62]) and the exactTest() function. Significantly deregulated genes were analyzed using STRING (https://string‐db.org/) [63]. For overrepresentation analysis, GO, KEGG pathway, and Reactome gene sets were considered.

LCK/PPARG signaling correlation scores were calculated by assigning a score to each comparison (Table 1) and combining these scores according to the indicated equation:

$$\begin{array}{ccc} & & \mathrm{LCK}/\mathrm{PPARG}\;\mathrm{correlation}\;\mathrm{score}\;=\frac{\mathrm{score}\left(a\right)+\mathrm{score}\left(b\right)}{2}\\ & & \;+\;\mathrm{score}\left(c\right)-\mathrm{score}\left(d\right)-\frac{\mathrm{score}\left(e\right)+\mathrm{score}\left(f\right)}{2} \end{array}$$

where a—DMSO_siLCK1 versus DMSO_siCtrl; b—DMSO_siLCK2 versus DMSO_siCtrl; c—GW_siCtrl versus DMSO_siCtrl; d—Rosi_siCtrl versus DMSO_siCtrl; e—Rosi_siLCK1 versus DMSO_siLCK1; f—Rosi_siLCK2 versus DMSO_siLCK2. (DMSO = dimethyl sulfoxide; GW = GW9662, Rosi = rosiglitazone)

**TABLE 1:** Score annotation for signaling correlation score calculation.

		log_2_(FC)				
Score		> 1	> 0.2	≥ 0.2 ∩ ≤ 0.2	< −0.2	< −1
FDR	≤ 0.05	3	1	0	−1	−3
	> 0.05	0.6	0.2	0	−0.2	−0.6

### 4.5 Gene Knockdown Using siRNAs

For transient LCK knockdown, 30 pmol siRNAs and 6 µL RNAiMax transfection reagent (Thermo Fisher Scientific) were diluted in Opti‐MEM (Thermo Fisher Scientific), mixed, and incubated for 5 min in 6‐well plates before addition of 6 × 10^5^ cells. After 24 h, 8 × 10^5^ cells were seeded for Western blotting, 4 × 10^5^ for RNA or lipid extraction, and 8 × 10^4^ for immunofluorescence.

LCK‐targeting siRNAs were siLCK1 (5′‐UUCGUAGGUAACCAGUGGGdTdT‐3′) and siLCK2 (5′‐UUUCCAUCCAGUCAUCUUCdTdT‐3′). AllStars siRNA (Qiagen, Hilden, Germany) served as non‐targeting control.

### 4.6 Protein Isolation and Western Blotting

Cells were gently harvested using a rubber policeman, washed with phosphate‐buffered saline (PBS), and lysed in total lysis buffer (50 mmol/L Tris‐HCl pH 7.4, 50 mmol/L NaCl, 2 mmol/L MgCl_2_, 1% (w/v) SDS) supplemented with protease inhibitors (Roche). Proteins were separated by SDS‐PAGE, transferred to nitrocellulose membranes (Th. Geyer, Renningen, Germany), and detected using a ChemiDoc MP Imaging System (BioRad, Hercules, CA, USA). Primary antibodies against LCK (clone 73A5, #2787), PPARG (clone C26H12, #2435), histone H3 (clone D1H2, #4499), H3K27ac (clone D5E4, #8173), p300 (clone E8S2V, #57625), alpha‐tubulin (clone DM1A, #3873), GAPDH (clone D16H11, #5174), and PARP (clone 46D11, #9532; all Cell Signaling Technology, Danvers, MA, USA) were used. Ponceau S staining served as loading control.

### 4.7 Subcellular Fractionation

Cells grown on 10‐cm plates were harvested, pelleted (500 × g, 3 min, 4°C), washed in PBS, pelleted again (800 × g, 5 min, 4°C), and resuspended in reticulocyte standard buffer (RSB; 10 mmol/L Tris‐HCl pH 7.4, 10 mmol/L NaCl, 3 mmol/L MgCl_2_). After 3 min on ice, cells were pelleted (1000 × g, 5 min, 4°C) and resuspended in RSBG40 (RSB supplemented with 0.5 mmol/L DTT, 10% glycerol, and 0.5% NP‐40) at four pellet volumes. Following 3 min on ice, samples were centrifuged at 4500 × g for 3 min at 4°C. The supernatant was collected as the CF. The remaining pellet was washed with RSBG40, incubated for 5 min on ice, and centrifuged at 9300 × g for 5 min at 4°C. The NF was lysed in total lysis buffer supplemented with Turbonuclease and EDTA‐free protease and phosphatase inhibitors (Roche). For Western blotting, fractions were mixed with 4× SDS sample buffer containing β‐mercaptoethanol.

### 4.8 Chromatin Immunoprecipitation‐Sequencing

Cells (1.2 × 10^7^) were seeded in four 15‐cm plates; two were treated with 500 nM LCK inhibitor A‐770041 and two with the corresponding volume of DMSO for 24 h. ChIP was performed using the SimpleChIP Enzymatic Chromatin IP Kit (#9003, Cell Signaling Technology) according to the manufacturer's instructions. Two percent chromatin input DNA and chromatin DNA enriched with the H3K27ac antibody (#8173, Cell Signaling Technology) were processed and sequenced by Genewiz. Reads were adapter‐ and quality‐trimmed using Cutadapt [56], aligned to UCSC hg38 with HiSat2 [57], and processed with Samtools [58]. FeatureCounts [59] was used for read summarization. Peaks were called using MACS3 [64] and annotated and compared using R/ChIPseeker [65].

### 4.9 Three‐Dimensional Proliferation and Matrigel‐Based Invasion Assay

For 3D proliferation and invasion assays, 1 × 10^3^ cells were seeded in 50 µL medium in ultra‐low attachment 96‐well plates (Corning). Plates were centrifuged at 300 × g for 5 min and incubated overnight. For invasion assays, 50 µL Matrigel (Corning) was added and spheres centrifuged at 300 × g for 5 min at 4°C. After Matrigel solidification for 30 min at 37°C, wells were filled to 200 µL with medium. Images were acquired immediately and at indicated time points using an IncuCyte S3 (Essen Bioscience, Goettingen, Germany).

### 4.10 Viability Assay

Cell viability was assessed by MTT (Merck, Darmstadt, Germany) as previously described [28]. Briefly, SAS and PaTu8988T cells (2 × 10^3^) or PaTu8902 and UPCI‐SSC‐084 (4 × 10^3^) cells were each seeded in 96‐well plates and treated with CAY10566 (0.01–1 µM) alone or combined with 50 µM rosiglitazone. After 48 h, cells were incubated with 1 mg/mL MTT for 3 h. Formazan precipitates were dissolved in DMSO, and absorbance was measured at 565 nm using a Tecan Infinite 200 Pro plate reader (Tecan, Männedorf, Switzerland).

### 4.11 Analysis of Lipid Droplets

Cells (8 × 10^4^) grown on coverslips were treated with 125 µM oleic acid for 24 h and subsequently stained with 1 µg/mL BODIPY 558/568 C12 (Thermo Fisher Scientific) for 10 min. After PBS washing, cells were fixed with 4% paraformaldehyde in PBS (pH 6.9, Carl Roth) for 30 min at room temperature. After PBS washing, images were acquired using a Nanolive 3D Cell Explorer‐Fluo (Nanolive, Tolochenaz, Switzerland) and analyzed using with ImageJ [66]. BODIPY intensity was normalized to the refraction index (RI), which correlates with cell area. Grubb's test was applied to identify and exclude outliers.

### 4.12 Lipidomics

Cells (4 × 10^5^) were grown for 24 h in 6‐well plates, gently scraped in PBS, pelleted and resuspended in 150 µL water. Methanol (200 µL), chloroform (400 µL), and 1 M HCl (15 µL) were added, followed by vortexing and shaking for 1 h at 550 rpm. After centrifugation (2000 g, 10 min), the organic phase was collected. The aqueous phase was re‐extracted with 100 µL methanol, 200 µL chloroform, and 10 µL 1 M HCl. Combined organic phases were dried under a nitrogen stream and dissolved in 50 µL dichloromethane. Untargeted lipidomics was performed in positive and negative polarity modes on a Bruker timsTOF Pro with VIP‐HESI source (Bruker Corporation, Billerica, MA, USA). Mass spectrometry (MS) was operated in PASEF mode (collision energy 30 eV; full scan 100–2200 *m/z*; two PASEF ramps; 0.32 s cycle time). The employed frontend was a Thermo Fisher Scientific Vanquish H UHPLC with a Waters Acquity BEH C18 column (150 mm × 1 mm × 1.7 µm; Waters Corporation, Milford, MA, USA). Mobile Phase A comprised 60% acetonitrile in aqueous 10 mmol/L ammonium formate, and mobile Phase B 10% acetonitrile in isopropanol with 10 mmol/L ammonium formate. The following gradient was employed at a constant flow rate of 70 µL/min and 40°C: 0 min; 2% B, 7 min; 2% B, 20 min; 100% B, 25 min; 100% B, followed by 5 min re‐equilibration to starting conditions. Data were analyzed using Bruker Compass MetaboScape 2022b Version 9.0.1 (Build 11878). Features were annotated using the rule‐based MCube LipidSpecies annotation algorithm and the MS‐DIAL LipidBlast spectral database v68.

### 4.13 Analysis of Lipid Peroxidation

Cells (2 × 10^4^) were seeded in 96‐well plates and, after 24 h, stained with 1 µM BODIPY 581/591 C11 (Cell Signaling Technology) for 30 min. Cells were then treated with CAY10566 (0.1–10 µM) for 3 h. Images were acquired and analyzed using an IncuCyte S3 (Essen Bioscience). Four images per well were analyzed, and mean fluorescence intensities of the green (510 nm, oxidized) and red (591 nm, non‐oxidized) channels were background‐corrected using untreated wells. The green/red ratio was calculated and normalized to the DMSO control.

### 4.14 Correlation of SCD Expression With Clinical Data

SCD expression in the TCGA HNSCC dataset and HNSCC subtypes, as well as its association with patient survival, were analyzed using the GEPIA2 portal [33].

### 4.15 Statistical Analysis

Statistical analyses were performed using GraphPad Prism 8.0 (GraphPad Software, San Diego, CA, USA). Student's *t*‐test was used for normally distributed two‐group comparisons, and the Mann–Whitney *U* test was used otherwise. One‐way ANOVA was used for multiple‐group comparisons, and two‐way ANOVA was used for grouped data. Dunnett's correction was used to correct for multiple testing. Differences were considered significant at *p* ≤ 0.05 (^*^ *p* ≤ 0.05, ^**^ *p* ≤ 0.01, ^***^ *p* ≤ 0.001).

## Author Contributions

J.P., T.T., M.H., R.B.‐G., and T.G. conceptualized the study. J.P., L.M., J.M., T.T., and M.S. performed the experiments. J.P., T.T., M.G., M.H., and T.G. analyzed the experimental data. J.P. and T.G. wrote the manuscript with input from all authors. J.P. and T.G. prepared the figures. All authors have read and agreed to the final version of the manuscript.

## Funding

This work was funded in whole or in part by the Austrian Science Fund (FWF) (SFB Lipid Hydrolysis [10.55776/F73]) to R.B.‐G. and T.T., and by intramural funding from the Medical Faculty Halle (Wilhelm‐Roux program, FKZ32/19) to T.G. For open access purposes, the authors have applied a CC BY public copyright license to any author‐accepted manuscript version arising from this submission.

## Ethics Statement

The authors have nothing to report.

## Conflicts of Interest

The authors declare no conflicts of interest.

## Supporting information




**Supporting File 1**: mco271060‐sup‐0001‐SuppMat.docx

**Supporting File 2**: mco271060‐sup‐0002‐Tables S1‐S3.xlsx

## Data Availability

The data underlying this article are available in the manuscript and its online Supporting Information. The raw RNA‐sequencing data can be downloaded from the GEO database (GSE342577). Metabolomics/Lipidomics data have been uploaded to the TU Wien Research Data Repository and can be freely accessed here: https://doi.org/10.48436/qs62v‐dhb46.
