# Supplementary material for: An LCK/PPARG/SCD Lipid Metabolism Axis Promotes Invasion in Human Oral Cancer
Source: MedComm (2020). 2026 Oct 13;7(10):e71060. doi: 10.1002/mco2.71060 (PMC13659266; doi:10.1002/mco2.71060)
Supplement: Supplementary file 1 [file MCO2-7-e71060-s001.docx]

**Supplementary Information**

**An LCK/PPARG/SCD Lipid Metabolism Axis Promotes Invasion in Human Oral Cancer**

**Running title:** LCK/PPARG/SCD Axis Promotes Invasion

Jonas Pyko^1^, Tamara Tomin^2^, Markus Glaß^3^, Lisa Müller^1^, Jana Macho^1^, Matthias Schittmayer^2^, Monika Haemmerle^4^, Ruth Birner-Gruenberger^2,5,*^, Tony Gutschner^1,*^

^1^Institute of Molecular Medicine, Section for RNA Biology and Pathogenesis, Faculty of Medicine, Martin Luther University Halle-Wittenberg, 06120 Halle (Saale), Germany

^2^Institute of Chemical Technologies and Analytics, Section for Bioanalytics, Faculty of Technical Chemistry, TU Wien, Vienna, Austria

^3^Institute of Molecular Medicine, Section for Molecular Cell Biology, Faculty of Medicine, Martin Luther University Halle-Wittenberg, 06120 Halle (Saale), Germany

^4^Institute of Pathology, Section for Experimental Pathology, Faculty of Medicine, Martin Luther University Halle-Wittenberg, 06120 Halle (Saale), Germany

^5^Diagnostic and Research institute of Pathology, Medical University of Graz, Graz, Austria.

***Corresponding authors:**

Ruth Birner-Gruenberger (RBG), E-Mail: ruth.birner-gruenberger@tuwien.ac.at,

and

Tony Gutschner (TG), E-Mail: tony.gutschner@uk-halle.de,

**This file contains:	I) Supplementary Figures S1 - S4**

**II) Legends for Supplementary Tables S1 - S3**

**I) Supplementary Figures**

**Figure S1. LCK inhibition reduces PPARG protein expression and global histone H3K27 acetylation.**

**(A)** Western blot analysis showing reduced LCK and PPARG protein expression after pharmacological inhibition of LCK. **(B)** Total histone H3 and acetylated H3K27 levels in SAS cell treated with LCK inhibitor A-770041 (LCKi). Ponceau S staining was used to assess overall protein loading in different samples. **(C)** Western blot showing intracellular localization of LCK in the nucleus and cytoplasm of SAS cells. The specificity of the LCK signal was confirmed using LCK knockdown cells, while PARP and GAPDH verified the purity of the nuclear and cytoplasmic fractions, respectively. **(D)** Western blot analysis after LCK knockdown shows that p300 expression remains unchanged. All western blots show representative results of three biological replicates.

**Figure S2. Effects of LCK or PPARG modulation on cell growth and invasion.**

Spheroid growth and relative invasiveness of SAS and UPCI-SSC-084 (oral cancer), Chl-1 (melanoma), PaTu 8902, and PaTu-8898T (pancreatic cancer) cells treated with 200 µM rosiglitazone or 500 nM LCKi. Representative pictures are shown (n = 3; scale bar = 800 µm).

**Figure S3. Phospholipid composition is altered in SAS clones and upon LCK inhibition.**

**(A)** Untargeted lipidomics comparing high invasive clones C, S and H with low invasive clones F, L and D. Features with C16 or C18 side chains were identified via tandem mass spectrometry (MS/MS). Features identified as significantly deregulated in LCK knockdown with parallel PPARG inhibition or activation were filtered for features with **(B)** Mono-unsaturated fatty acids (MUFAs), **(C)** Poly-unsaturated fatty acids (PUFAs), and **(D)** Saturated fatty acids (SFAs) identified by MS/MS. Two-way-Analysis of Variance (ANOVA) was performed to compare to siCtrl (#*P* ≤ 0.05, ##*P* ≤ 0.01, ###*P* ≤ 0.001) or DMSO treatment (**P* ≤ 0.05, ***P* ≤ 0.01, ****P* ≤ 0.001).

**Figure S4. SCD expression is increased in Head and Neck Squamous Cell Carcinoma (HNSCC) and correlates with patient survival.**

**(A)** Increase of *SCD* mRNA levels in indicated cell lines after rosiglitazone treatment (50 µM, 48 h). **(B)** Expression of *SCD* mRNA in HNSCC samples (The Cancer Genome Atlas (TCGA) cohort, red) compared to normal tissues (grey), and **(C)** Subtype-resolved expression of *SCD* in the same TCGA HNSCC cohort. **(D)** Kaplan-Meier curves for disease-free survival of HNSCC patients assigned to the indicated molecular subtypes and stratified by *SCD* mRNA expression. Samples were divided by median separation. All expression and survival data were retrieved from GEPIA2 portal (http://gepia2.cancer-pku.cn/#index, [37]).

**II) Legends for Supplementary Tables**

**Table S1. Differentially expressed genes in high vs. low invasive SAS clones.** Table contains 323 genes that show a significantly (FDR ≤ 0.05) alteration (|log2(FC)| ≥ 1) in the highly invasive clone S compared to the derived from the weakly invasive clone F. Both clones were extracted from Matrigel and gene expression was analyzed using RNA-sequencing.

**Table S2.** **Differential gene expression and H3K27ac ChIP-seq peak changes following LCK inhibitor treatment. Compiled data showing differentially expressed genes** (|log2(FC)| ≥ 1**; FDR ≤ 0.05) after 48 h of 500 nM LCKi treatment alongside corresponding changes in histone 3 lysine 27 acetylation (H3K27ac) peaks measured by ChIP-sequencing after 24 h of LCKi treatment.**

**Table S3. Differential gene expression under parallel LCK knockdown and PPARG modulation.** Compiled transcriptomic data filtering for genes with an LCK/PPARG correlation score ≥ 2.5 and at least one significant change (FDR ≤ 0.05) across LCK knockdown, 50 µM rosiglitazone treatment, and 20 µM GW9662 treatment conditions.
